# Field Evidence: Microbial Fertilizer Drives Rhizosphere Phosphorus Transformation and Acidity Regulation to Synergistically Promote Chlorogenic Acid Accumulation in *Lonicera macranthoides*

**DOI:** 10.3390/microorganisms14081816

**Published:** 2026-08-18

**Authors:** Yong Wang, Kuaifen Li, Huarong Qiu, Qiuju Jiang, Qian Ding, Tangyan Li, Hua Feng, Xianyu Deng

**Affiliations:** 1 Key Laboratory of Conservation, Development and Utilization of Karst Characteristic Biological Resources, Department of Modern Agriculture, Zunyi Vocational and Technical College, Zunyi 563006, China; wykarst@163.com (Y.W.); likuaifengzu@126.com (K.L.); qiu516@126.com (H.Q.); jiangqiuju1990@126.com (Q.J.);; 2 College of Landscape and Horticulture, Southwest Forestry University, Kunming 650224, China

**Keywords:** microbial fertilizer, phosphorus biotransformation, rhizosphere acidity regulation, chlorogenic acid accumulation, secondary metabolism

## Abstract

Microbial fertilizers may improve phosphorus availability and pH in acidic soils, but field evidence linking these changes with medicinal plant biomass and specialised metabolite accumulation remains limited. Here, a one-season field experiment was conducted in acidic yellow soil in Guizhou Province, China, using four fertilization regimes for *Lonicera macranthoides*: an organic fertilizer plus compound fertilizer control (CK), a bacterial consortium (T1), a simplified bacterial combination (T2), and a fungal agent (T3). Soil chemical properties, soil aggregate composition, flower-bud biomass, and chlorogenic-acid-related compounds were measured. T1 and T3 increased soil available phosphorus and pH at the pre-flowering stage and increased the proportion of water-stable macroaggregates (>5 mm). Both treatments also increased fresh and dry biomass. T1 showed the highest numerical chlorogenic acid content, whereas T3 was more favourable for the accumulation of isochlorogenic acids A and C. Across plot-level observations, available phosphorus was positively correlated with fresh weight (*r* = 0.804) and dry weight (*r* = 0.781), and pH was positively correlated with chlorogenic acid (*r* = 0.687). Univariate regression and redundancy analysis further indicated that available phosphorus and pH were the soil factors most closely associated with biomass and chlorogenic acid accumulation. These findings provide preliminary field indications that microbial fertilizers may improve yield and medicinal quality in acidic-soil *L. macranthoides* production. However, the single-season, single-site nature of the experiment warrants cautious interpretation and further validation across broader conditions. The observed associations are consistent with, but do not prove, a mechanistic pathway involving microbe-mediated phosphorus transformation and acidity regulation.

## 1. Introduction

Against the background of increasing global soil acidification and degradation of cultivated land resources, microbial fertilizers are widely used to ameliorate acidic soils and improve crop nutrient use efficiency [1,2]. However, significant differences exist among different functional microbial groups in terms of nutrient transformation pathways, rhizosphere regulation modes, and plant response mechanisms [3,4]. Current research remains primarily focused on yield effects and lacks a systematic investigation within an integrated framework of soil biogeochemical processes, rhizosphere environment regulation, and plant metabolic responses. In acidic ecosystems, soil phosphorus often exists in iron- or aluminum-bound forms or as insoluble minerals, severely limiting plant uptake [5,6]. Functional microorganisms such as Bacillus and arbuscular mycorrhizal fungi can promote the conversion of phosphorus into labile pools by secreting organic acids and phosphatases and by extending the rhizosphere interface [7,8]. However, most existing studies have been conducted under laboratory or short-term pot conditions; systematic quantitative evidence regarding the dynamics of microbe-mediated phosphorus transformation and its regulatory effects on plant metabolism under field conditions remains limited.

In addition to nutrient transformation, the rhizosphere acidity environment is an important regulatory factor affecting microbial functional expression and the chemical forms of nutrients. In acidic soils, increased activity of Fe^3+^ and Al^3+^ tends to exacerbate phosphorus fixation and inhibit microbial enzyme activity, whereas an increase in pH can significantly improve phosphorus desorption and promote microbial metabolic activity [9]. Microbial inoculants not only function through direct participation in nutrient transformation but also indirectly affect nutrient availability and plant uptake by altering rhizosphere pH and buffering acidification stress [10]. Nevertheless, systematic research on whether microbe-mediated acidity regulation synergistically drives changes in plant secondary metabolism together with phosphorus biotransformation processes is still lacking [11].

*Lonicera macranthoides* is a medicinal plant typically rich in chlorogenic acid compounds [12]. The synthesis of its active components relies mainly on the phenylpropanoid metabolic pathway and is highly sensitive to phosphorus supply and the rhizosphere nutrient environment [13]. Chlorogenic acid and its isomers not only determine medicinal quality but also reflect the metabolic response of plants to environmental nutrient conditions [14]. Previous studies have indicated that nutrient supply levels can significantly affect chlorogenic acid accumulation. However, most work has focused on the effects of chemical fertilizer application, leaving a clear gap in research on how microbe-driven soil processes regulate the allocation of chlorogenic acid metabolism [15,16,17].

The microbial inoculants used in this study (*Bacillus*, *Paenibacillus*, *Trichoderma*, and arbuscular mycorrhizal fungi) were selected for their complementary functional traits in phosphorus solubilization (organic acid and phosphatase secretion) and rhizosphere acidification regulation. Phosphorus supply and pH can influence the phenylpropanoid pathway by modulating substrate availability, ATP supply, and micronutrient cofactor bioavailability for key enzymes such as phenylalanine ammonia-lyase [18,19]. However, field-based quantitative evidence linking these microbe-driven processes to chlorogenic acid accumulation in medicinal plants remains scarce, and the differential effects of bacterial consortia versus fungal agents on specific chlorogenic acid isomers have not been systematically examined. To address these gaps, we conducted a one-season field experiment in a typical acidic yellow-soil region of Guizhou Province with four fertilization regimes.

This study used the *L. macranthoides* cultivation system in a typical acidic soil region of Guizhou Province to systematically compare the effects of different types of microbial inoculants on soil nutrient dynamics, rhizosphere acidity changes, biomass formation, and chlorogenic acid component accumulation. The focus was to elucidate the mechanisms by which microbe-mediated phosphorus biotransformation and acidity regulation control plant secondary metabolism. The hypotheses of this study are as follows: (1) microbial inoculants can significantly enhance the efficiency of phosphorus biotransformation in acidic soils and improve the rhizosphere acidity environment; (2) phosphorus availability and pH changes are key regulatory factors driving chlorogenic acid accumulation in *L. macranthoides*; and (3) different functional microbial communities influence soil processes and plant metabolic responses via distinct pathways. The findings aim to reveal, at the field scale, the mechanisms by which microbe-driven soil biogeochemical processes regulate the quality formation of medicinal plants, thereby providing a theoretical basis for the precise application of microbial fertilizers in acidic ecosystems.

## 2. Materials and Methods

### 2.1. Site Description and Experimental Design

**Figure 1:**
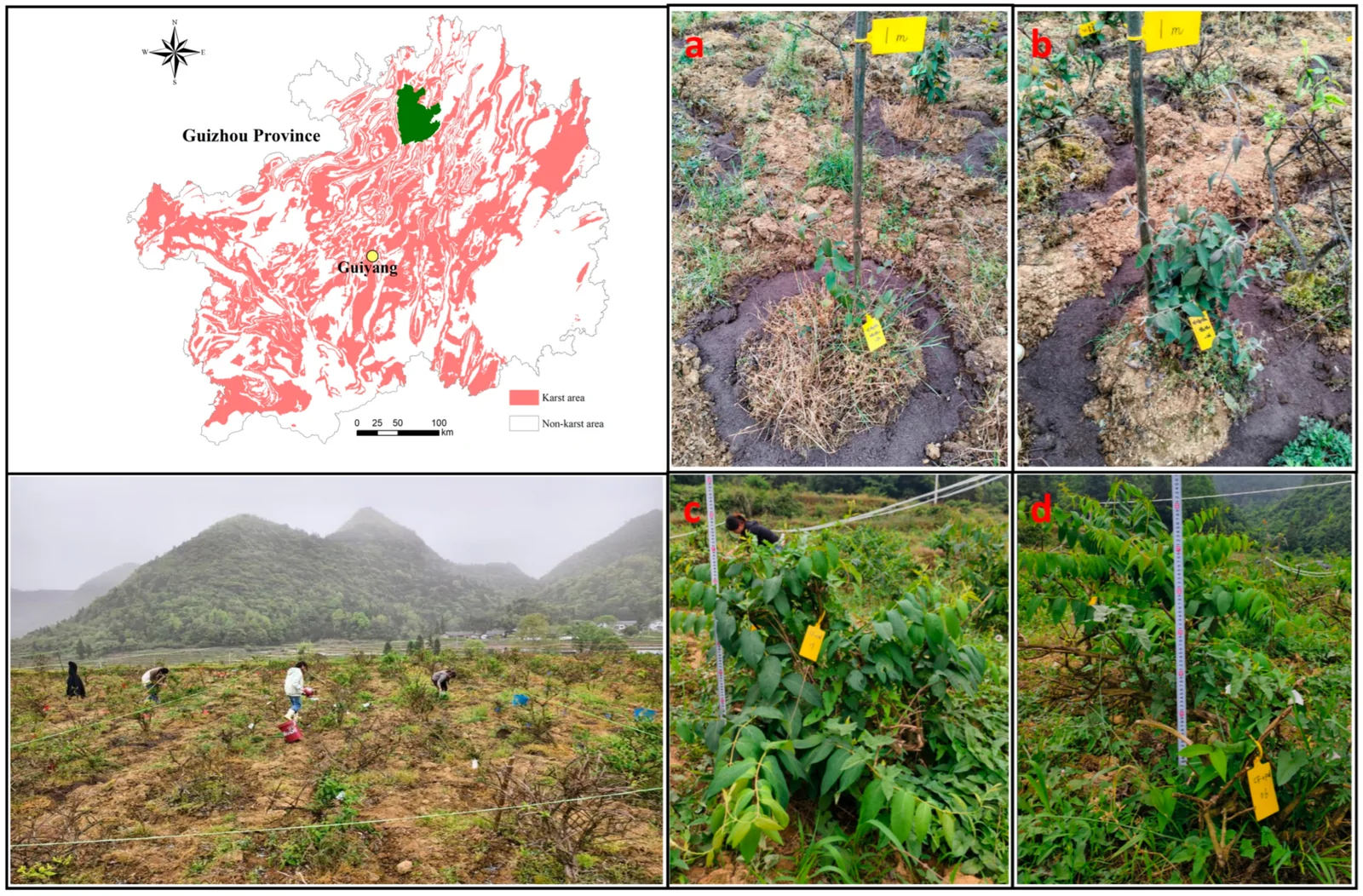
Overview of the study area. **(a,b) Before fertilization; (c,d) After fertilization.**

The field experiment was conducted in 2025 in a typical *Lonicera macranthoides* planting area at Fengkanping, Fenghua Town, Suiyang County, Zunyi City, Guizhou Province, China. The geographic coordinates of the experimental site were 107°07′09″ E, 28°01′10″ N, at an altitude of 1005 m (Figure 1). The test crop was five-year-old *Lonicera macranthoides*. The soil at the site was acidic yellow soil, with an initial pH ranging from 4.38 to 4.92. The experimental site was selected because it represents the typical acidic yellow-soil conditions under which *L. macranthoides* is commercially cultivated in the Guizhou highlands. The five-year-old plants were established and managed following local agronomic practices, ensuring that the trial conditions were representative of real-world production systems. No prior field experiments with microbial consortia had been conducted at this specific site; however, the fertilization regimes (organic + compound fertilizer) reflected standard local practice, against which the microbial treatments could be meaningfully compared.

Four fertilization treatments were established with three replicate field plots per treatment (12 plots in total). Each replicate plot contained 150 plants and was treated as the independent experimental unit for statistical analysis. Soil subsamples and plant measurements within a plot were composited or averaged before treatment-level comparisons. The treatments were as follows:

CK: organic fertilizer (4 kg per plant) + compound fertilizer (100 g per plant);
T1: organic fertilizer (4 kg per plant) + compound fertilizer (100 g per plant) + bacterial consortium (25 mL per plant);
T2: based on T1, the bacterial consortium was replaced with a simplified bacterial combination (25 mL per plant);
T3: based on T1, the bacterial consortium was replaced with a fungal agent (25 mL per plant).

The bacterial consortium was prepared by mixing equal volumes of *Paenibacillus polymyxa* ACCC 10248, *Bacillus amyloliquefaciens* ACCC 11025, *Bacillus subtilis* ACCC 11089, *Bacillus velezensis* ACCC 11067, and *Bacillus nematocida* ACCC 11078, with each strain at a concentration of 10^8^ cfu mL^−1^. The simplified bacterial combination consisted of *Paenibacillus polymyxa* ACCC 10248, *Bacillus amyloliquefaciens* ACCC 11025, and *Bacillus subtilis* ACCC 11089, mixed in equal volume proportions. The fungal agent was composed of *Arbuscular mycorrhiza* ACCC 60001, *Trichoderma harzianum* ACCC 30371, and *Trichoderma longibrachiatum* ACCC 30372, at a concentration of 10^8^ cfu mL^−1^. All microbial strains were provided by Guizhou Zhongke Weinong Biotechnology Co., Ltd. (Zunyi, China), and were originally sourced from the Agricultural Culture Collection of China (ACCC). The strains were cultivated according to the supplier’s recommended media and conditions prior to field application.

All fertilizers and microbial agents were applied once as basal fertilizer on 26 March 2025. The microbial agents were thoroughly mixed with organic fertilizer and applied in furrows at the plant rhizosphere, then covered with soil to ensure full contact between the agents and the rhizosphere soil.

### 2.2. Plot Establishment, Soil Collection, and Sample Analysis

#### 2.2.1. Soil Sampling and Chemical Property Analysis

Soil samples were collected before fertilization (15 March 2025), at the pre-flowering stage (1 May 2025), and after harvest (1 July 2025); the basal fertilizer was applied on 26 March 2025. Within each experimental plot, an S-shaped sampling pattern was used, and 5–8 sampling points were selected within the canopy projection area, corresponding to the concentrated root zone of *L. macranthoides*. At each point, the surface 3 mm of soil was scraped away, and a soil column from the 0–20 cm plough layer was taken vertically using a soil auger. Soil from all sampling points within the same plot was thoroughly mixed and then crushed by hand. The quartering method was used to retain approximately 1.5 kg of soil in a labelled sample bag. The mixed sample from each plot at each date was used as one analytical replicate.

After air-drying naturally, the samples were removed of plant debris and gravel, ground, and passed through 1 mm and 0.15 mm sieves for subsequent analyses. The detailed methods for each indicator are as follows:

(1) Soil pH: determined by the potentiometric method (soil:water = 1:2.5) using a PHS-3C pH meter.
(2) Soil organic carbon (SOC): determined by the potassium dichromate external heating method [NY/T 1121.6-2006] [20].
(3) Soil total nitrogen (TN): determined by the semi-micro Kjeldahl method [NY/T 53-1987] [21].
(4) Soil hydrolyzable nitrogen [7]: Determined by the alkaline diffusion method. Air-dried soil (2.00 g) passing through a 1 mm sieve was placed in the outer chamber of a diffusion dish, and 10 mL of 1.0 mol L^−1^ NaOH solution was added, while the inner chamber received 2 mL of 2% boric acid solution. The dish was sealed and placed in an incubator at 40 °C for 24 h. After incubation, the inner chamber was titrated with 0.01 mol L^−1^ HCl standard solution until a purplish-red color appeared. A blank test was run simultaneously. The hydrolyzable nitrogen content was expressed as alkaline hydrolyzable nitrogen.
(5) Soil total phosphorus: Determined by the NaOH fusion—Mo-Sb colorimetric method. Air-dried soil (0.25 g) passing through a 0.15 mm sieve was placed in a nickel crucible, and 2 g of NaOH was added. The crucible was heated in a muffle furnace at 720 °C for 15 min. After cooling, the melt was extracted with hot water and acidified with concentrated HCl to pH ≈ 8, then diluted to 100 mL. An aliquot of the solution was taken, Mo-Sb color developer was added, and the absorbance was measured at 700 nm using a UV-Vis spectrophotometer. The total phosphorus content was calculated from a standard curve.
(6) Soil available phosphorus (AP): Determined by the 0.5 mol L^−1^ NaHCO_3_ extraction—Mo-Sb colorimetric method (Olsen method). Air-dried soil (2.50 g) passing through a 1 mm sieve was placed in a 150 mL conical flask, and 50 mL of 0.5 mol L^−1^ NaHCO_3_ solution (pH 8.5) was added. The mixture was shaken for 30 min and filtered. A 10 mL aliquot of the filtrate was transferred to a 50 mL volumetric flask, 5 mL of Mo-Sb color developer was added, and the volume was made up with water. The absorbance was measured at 700 nm, and the available phosphorus content was calculated from a standard curve.
(7) Soil total potassium: Determined by the NaOH fusion—flame photometric method. The fusion procedure was the same as that for total phosphorus. After fusion, the extract was analyzed for potassium emission intensity using a flame photometer, and the total potassium content was calculated from a standard curve.
(8) Soil available potassium [4]: Determined by the 1 mol L^−1^ ammonium acetate extraction—flame photometric method. Air-dried soil (5.00 g) passing through a 1 mm sieve was placed in a 100 mL conical flask, and 50 mL of 1 mol L^−1^ ammonium acetate solution (pH 7.0) was added. The mixture was shaken for 30 min and filtered. The filtrate was directly measured with a flame photometer, and the available potassium content was calculated from a standard curve.
(9) Water-stable soil aggregates: Determined by the wet sieving method. Air-dried soil (50.00 g) passing through an 8 mm sieve was placed on the top sieve (5 mm) of an aggregate analyzer, with the following stack of sieves: 5 mm, 2 mm, 1 mm, 0.5 mm, 0.25 mm, and 0.15 mm. Deionized water was added, and the aggregates were wet sieved for 5 min at a shaking frequency of 30 strokes per minute. The aggregates retained on each sieve were rinsed into aluminum boxes, dried at 50 °C, and weighed. The mass fraction of each aggregate size class (%) was calculated as (dry weight of aggregates in that class/total dry weight) × 100%. Size classes were: >5 mm, 2–5 mm, 1–2 mm, 0.5–1 mm, 0.25–0.5 mm, 0.15–0.25 mm, and <0.15 mm.
(10) Non-water-stable soil aggregates: Determined by the dry sieving method. Air-dried soil (200.00 g) passing through an 8 mm sieve was placed on the top sieve of a stack (5 mm, 2 mm, 1 mm, 0.5 mm, 0.25 mm, 0.15 mm) and sieved on an oscillating shaker for 5 min (amplitude 4 mm, frequency 300 Hz). The soil retained on each sieve was weighed, and the mass fraction for each size class was calculated. Size classes were the same as for water-stable aggregates.

#### 2.2.2. Determination of *Lonicera macranthoides* Yield and Active Components

(1) Biomass determination: At the full flowering stage on 1 July 2025, flower buds were collected in each experimental plot following the method described above. In each replicate plot of each treatment, 30 plants were randomly selected, and all flower buds were harvested separately. The fresh weight of buds per plant was immediately measured using an electronic balance (accuracy 0.01 g), and plant-level values were averaged to obtain one plot-level value for statistical analysis. After weighing, the buds were steam-treated at 60 °C for 5 min in a steam deactivation machine to inactivate oxidase activity. The deactivated samples were then transferred to a constant-temperature forced-air drying oven and dried at 50 °C to constant weight (the difference between two consecutive weighings less than 0.005 g), and the dry weight per plant was determined. The dry-to-fresh ratio (%) was calculated as dry weight divided by fresh weight multiplied by 100.
(2) Extraction of active components: The dried flower buds of *L. macranthoides* were ground in a high-speed grinder and passed through a 60-mesh sieve (0.25 mm aperture). The fine powder was collected. Accurately weighed 1.00 g (to the nearest 0.0001 g) of the powder was placed in a 50 mL stoppered conical flask, and 50 mL of 70% methanol (chromatographic grade methanol:deionized water = 70:30, *v*/*v*) was added at a solid-to-liquid ratio of 1:50 (g·mL^−1^). The mixture was allowed to stand overnight in the dark (≥12 h) and then ultrasonically extracted at room temperature for 30 min (power 200 W, frequency 40 kHz). The extract was centrifuged at 4000 r·min^−1^ for 10 min, and the supernatant was filtered through a 0.22 μm polytetrafluoroethylene (PTFE) membrane. The filtrate was transferred to a 2 mL brown glass vial, sealed, and stored at 4 °C in the dark until HPLC analysis.
(3) High-performance liquid chromatography (HPLC) conditions: refer to the method by Nguyen et al. [21]. In brief, the HPLC system was a Shimadzu LC-2030 equipped with a diode array detector. The column was an Agilent ZORBAX Eclipse Plus C18 column (4.6 mm × 250 mm, 5 μm). The column temperature was 30 °C. The detection wavelength was 265 nm, and full-wavelength scanning from 200 to 400 nm was simultaneously performed for peak purity confirmation. Mobile phase A was 0.1% phosphoric acid in water, and mobile phase B was acetonitrile (chromatographic grade). The gradient elution program was as follows: at 0 min, 10% B; held at 10% B for 4 min; from 4 to 15.5 min, B increased linearly from 10% to 21.8%; from 15.5 to 24.6 min, B increased linearly from 21.8% to 25%; from 24.6 to 26.3 min, B increased linearly from 25% to 60%; from 26.3 to 30.2 min, B decreased linearly from 60% to 5%; from 30.2 to 35.2 min, B was held at 5% for post-equilibration (5 min), and finally returned to 10% B. The flow rate was 1.0 mL·min^−1^, and the injection volume was 10 μL.
(4) Quantitative analysis: The external standard method was used for quantification. The analytes included chlorogenic acid, neochlorogenic acid, cryptochlorogenic acid, isochlorogenic acid A, isochlorogenic acid B, isochlorogenic acid C, isoquercitrin, and astragalin. The content of each component in the samples was calculated as follows:
  Content (mg·g^−1^) = (C × V × D)/(m × 1000)
  where C is the concentration of the test solution obtained from the standard curve (μg·mL^−1^), V is the volume of the extraction solvent (mL), D is the dilution factor (1 for undiluted samples), and m is the sample mass (g). Each sample was injected twice, and the average value was taken. Final results are expressed as mean ± standard deviation (*n* = 3).
(5) Method validation: The accuracy of the method was evaluated by spiked recovery tests. A 0.5 g portion of sample powder with known concentrations was spiked with mixed standards at three levels (50%, 100%, and 150% of the original content), processed according to the extraction and determination procedure described above, and the recovery of each component was calculated (expected recovery between 90% and 110%). For precision, the same standard solution was injected six times consecutively, and the relative standard deviation (RSD) of peak areas was calculated (expected RSD < 2%). For repeatability, six independent sample preparations from the same batch were extracted and measured, and the RSD of the content values was calculated. The limit of detection (LOD) and limit of quantification (LOQ) were determined based on signal-to-noise ratios (S/N) of 3 and 10, respectively.

### 2.3. Data Analysis

Data organisation and basic statistical analyses were performed using Excel 2024 and SPSS 2022. For soil chemical properties and aggregate fractions measured across sampling dates, treatments, sampling times, and their interactions, two-way analysis of variance was used. When treatment effects were examined within a sampling date, Duncan’s multiple range test was used for mean separation (*p* < 0.05). Biomass and active-component contents measured at harvest were analysed by one-way analysis of variance followed by Duncan’s test (*p* < 0.05). The replicate field plot was used as the independent experimental unit (*n* = 3 per treatment). Pearson correlation analysis was performed on plot-level data to evaluate associations between soil nutrient indices and yield or active components. Because many soil and metabolite variables were tested, the univariate results were interpreted as exploratory and were emphasised only when supported by consistent patterns across ANOVA, correlation, regression, and RDA.

To describe the explanatory associations between soil factors and *L. macranthoides* yield or chlorogenic acid accumulation, univariate linear regression models were applied. Soil organic carbon (SOC), total nitrogen, total phosphorus, available phosphorus (AP), available potassium [4], and pH were used as independent variables, whereas fresh weight, dry weight, and total chlorogenic acid content were used as dependent variables. The regression equation y = a + bx was constructed, and the coefficient of determination (*R*^2^) and its significance were calculated. These models were used to identify statistical associations, not to infer direct microbial mechanisms or causality.

To visualise multivariate associations between soil factors and multiple response variables, redundancy analysis (RDA) was performed. Soil physicochemical factors (AP, pH, TP, and TK) were used as environmental variables, and the response variables were fresh weight, dry weight, chlorogenic acid, neochlorogenic acid, cryptochlorogenic acid, isochlorogenic acid A, isochlorogenic acid B, and isochlorogenic acid C. Constrained ordination was carried out using Canoco 5.0. The significance of the constrained axes was assessed by permutation tests (999 permutations). Graphs were created using OriginPro 2024 and the ggplot2 package in R.

## 3. Results

### 3.1. Temporal Dynamics of Soil Chemical Properties Under Different Microbial Fertilizer Treatments

As shown in Table 1, compared with CK, T1, T2, and T3 significantly increased soil organic carbon content in May. In July, organic carbon in T2 and T3 remained significantly higher than in the CK treatment. Soil total phosphorus peaked in May, with T1 and T3 significantly higher than CK. In July, only T3 showed significantly higher total phosphorus than CK. Available phosphorus in May was highest in T3, followed by T1 and T2, all significantly exceeding CK. In July, all microbial treatments maintained significantly higher available phosphorus levels than the CK treatment. Soil pH increased from CK to T1, T2, and T3 in May. In July, pH values in all microbial treatments remained significantly higher than in the CK treatment. In May, total nitrogen and hydrolyzable nitrogen in T1 and T3 were significantly higher than in CK, with T2 showing intermediate values. In July, total nitrogen in T3 was significantly higher than in all other treatments. Total potassium in May was highest in CK and lowest in T1. In July, total potassium was highest in T3. Available potassium in May was highest in T1, followed by T2 and T3; in July, available potassium was highest in T3.

### 3.2. Temporal Dynamics of Soil Chemical Properties and Water-Stable Aggregates Under Different Microbial Fertilizer Treatments

As shown in Table 2, compared with CK, T1, T2, and T3 significantly increased soil organic carbon, total phosphorus, available phosphorus, total nitrogen, hydrolyzable nitrogen, and pH in May, with T1 and T3 showing stronger effects than T2. In July, available phosphorus and pH in all microbial treatments remained significantly higher than in CK, and total nitrogen in T3 was significantly higher than in all other treatments. Total potassium in May was highest in CK and lowest in T1, while in July it was highest in T3. Available potassium in May was highest in T1, followed by T2 and T3, and in July it was highest in T3. Regarding soil aggregates, microbial treatments significantly increased the proportion of >5 mm aggregates and decreased the proportions of small fractions such as 0.25–0.5 mm and 0.15–0.25 mm. For the 2–5 mm fraction, all microbial treatments were significantly higher than CK in July. For the 1–2 mm fraction, CK was highest in May, while T1 and T2 recovered significantly in July. No significant differences were observed among treatments for the 0.5–1 mm fraction. For the <0.15 mm fraction, T2 and T3 were highest in May, whereas CK was highest in July.

### 3.3. Effects of Different Microbial Fertilizer Treatments on Soil Non-Water-Stable Aggregates

As shown in Table 3, regarding non-water-stable aggregates, the >5 mm fraction was significantly higher in T3 than in CK only in May, with no significant differences between T1 or T2 and CK, and no differences among treatments in July. No significant differences were observed among treatments for the 2–5 mm and 0.5–1 mm fractions at any sampling time. For the 1–2 mm fraction, CK and T2 were significantly higher than T1 in May, with no differences among treatments in July. For the 0.25–0.5 mm fraction, CK was significantly higher than T2 and T3 in May, while CK and T1 were significantly higher than T3 in July. No significant differences were observed among treatments for the 0.15–0.25 mm fraction at any sampling time. For the <0.15 mm fraction, T1 and T3 were higher than CK and T2 in May, but the differences were not significant, and no differences were observed among treatments in July.

### 3.4. Responses of Lonicera macranthoides Biomass and Chlorogenic Acid Components to Different Microbial Fertilizer Treatments

As shown in Table 4, for biomass, fresh weight and dry weight in T1 and T3 were significantly higher than in CK, with T1 reaching the highest levels among all treatments, while T2 showed no significant difference compared with CK. For chlorogenic acid components, chlorogenic acid content was highest in T1, followed by T3 and T2, though the difference did not reach statistical significance. Neochlorogenic acid was significantly higher in T2 and T3 than in CK. Cryptochlorogenic acid showed no significant differences among treatments. For isochlorogenic acids, isochlorogenic acid A was significantly higher in T1 and T3 than in CK and T2, with T1 showing the highest value. Isochlorogenic acid B showed no significant differences among treatments. Isochlorogenic acid C was significantly higher in T1 than in CK, with T2 and T3 showing intermediate levels. For flavonoids, isoquercitrin and astragalin showed no significant differences among treatments. Overall, T1 exhibited the strongest promotion effect on biomass accumulation and chlorogenic acid components, followed by T3, while T2 showed limited effects.

The superior performance of T1 over T2 in biomass accumulation suggests that the full bacterial consortium (five strains) was more effective than the simplified three-strain combination. This difference may reflect functional complementarity among the five component strains, particularly the presence of *B. velezensis* and *B. nematocida* in T1, which are known for their plant-growth-promoting and biocontrol traits. However, because the present study did not test each component strain individually, we cannot determine whether the enhanced effect of T1 arose from the additive action of multiple strains, synergistic interactions, or the dominant contribution of a single key strain.

### 3.5. Correlations and Regressions Between Soil Nutrients and Lonicera macranthoides Yield and Chlorogenic Acid Accumulation

**Figure 2:**
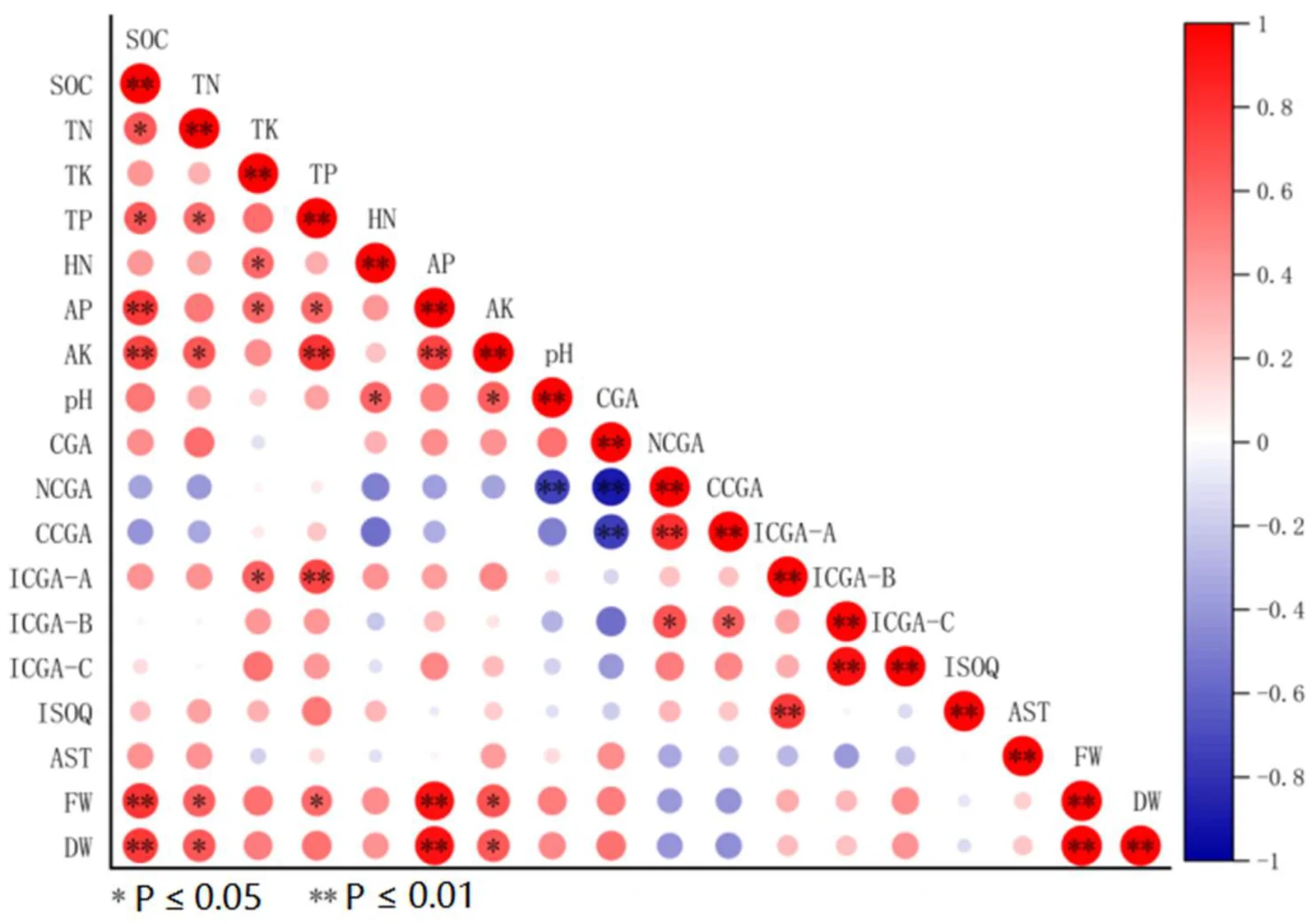
Correlation analysis between soil nutrients and the quality and yield of *Lonicera macranthoides*.

As shown in Table 5 and Figure 2, different fertilization treatments were significantly correlated with chlorogenic acid (*r* = 0.707, *p* < 0.05), neochlorogenic acid (*r* = −0.593, *p* < 0.05), cryptochlorogenic acid (*r* = −0.698, *p* < 0.05), fresh weight (*r* = 0.608, *p* < 0.05), and dry weight (*r* = 0.607, *p* < 0.05). Soil organic carbon and total nitrogen were significantly positively correlated with fresh weight (*r* = 0.603 and 0.612, *p* < 0.05) and dry weight (*r* = 0.604 and 0.619, *p* < 0.05). Total potassium and total phosphorus were significantly positively correlated with isochlorogenic acid A (*r* = 0.836 and 0.865, *p* < 0.01) and with isochlorogenic acid C (*r* = 0.600 and 0.624, *p* < 0.05). Available phosphorus was significantly positively correlated with fresh weight (*r* = 0.804, *p* < 0.01) and dry weight (*r* = 0.781, *p* < 0.01), and showed a positive but non-significant correlation with chlorogenic acid (*r* = 0.538, *p* > 0.05). pH was significantly positively correlated with chlorogenic acid (*r* = 0.687, *p* < 0.05), and significantly negatively correlated with neochlorogenic acid (*r* = −0.607, *p* < 0.05) and cryptochlorogenic acid (*r* = −0.681, *p* < 0.05). Hydrolyzable nitrogen and available potassium showed no significant correlations with any of the indicators. All correlation *p*-values reported are nominal and have not been adjusted for multiple comparisons. Given the relatively small sample size (*n* = 12 plots) and the number of variables tested, these significant associations should be interpreted as exploratory rather than confirmatory, and they are presented to identify candidate relationships for future targeted investigation.

The regression analysis results (Table 6) showed that different soil factors explained varying degrees of variation in *L. macranthoides* yield and chlorogenic acid accumulation. For fresh weight, available phosphorus had the highest explanatory power (_R_^2^ = 0.646, *p* = 0.002), followed by total nitrogen (_R_^2^ = 0.374, *p* = 0.035) and organic carbon (_R_^2^ = 0.364, *p* = 0.038). For dry weight, available phosphorus also had the highest explanatory power (_R_^2^ = 0.610, *p* = 0.003), followed by total nitrogen (_R_^2^ = 0.384, *p* = 0.032) and organic carbon (_R_^2^ = 0.365, *p* = 0.038). For total chlorogenic acid, available phosphorus had the strongest explanatory power (_R_^2^ = 0.727, *p* < 0.001), followed by pH (_R_^2^ = 0.512, *p* = 0.016), available potassium (_R_^2^ = 0.501, *p* = 0.010), organic carbon (_R_^2^ = 0.392, *p* = 0.029), total nitrogen (_R_^2^ = 0.370, *p* = 0.036), and total phosphorus (_R_^2^ = 0.346, *p* = 0.044).

### 3.6. Integrated Response Characteristics Under Different Microbial Fertilizer Treatments

**Figure 3:**
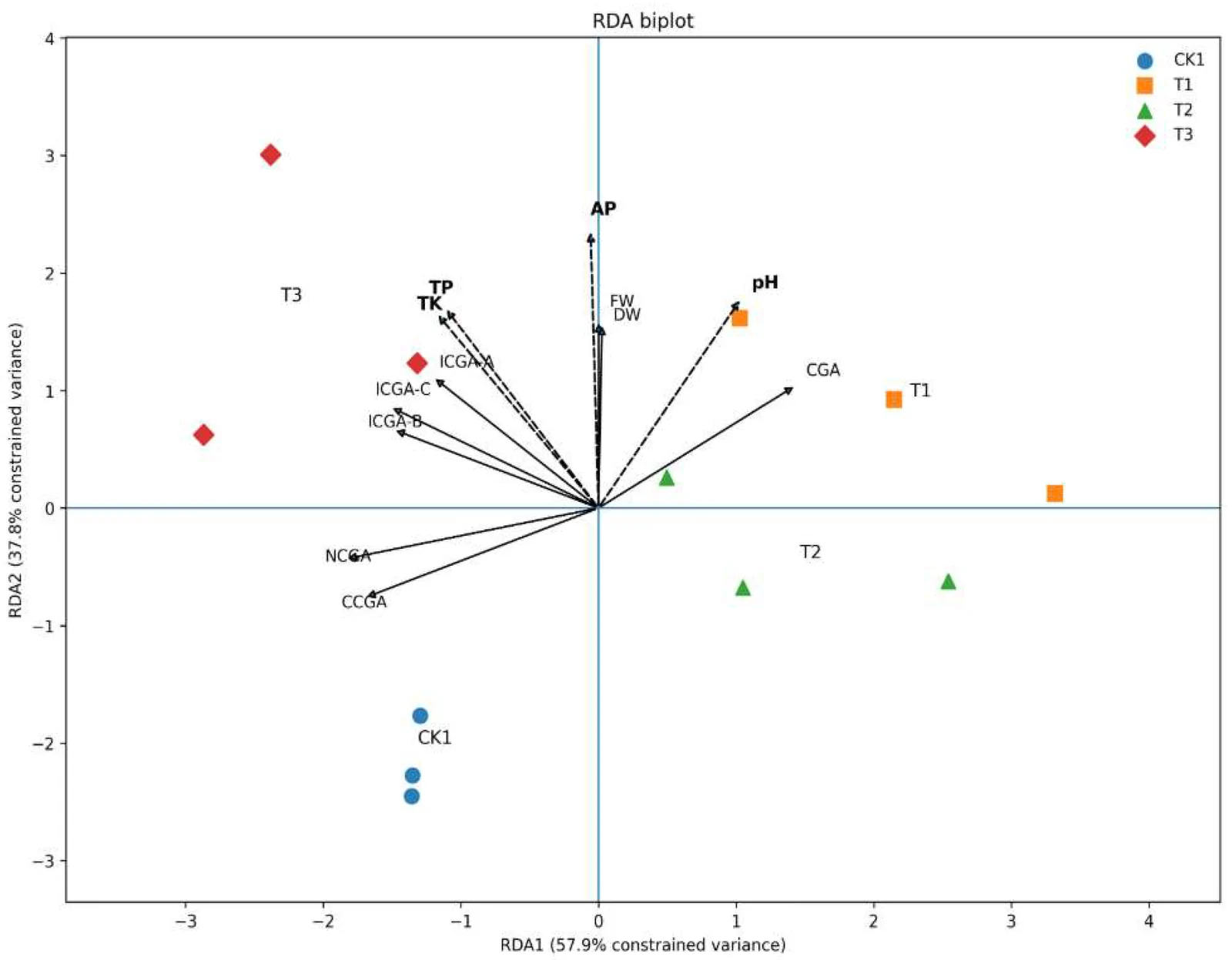
Redundancy analysis (RDA) biplot showing the relationships among soil properties, biomass, and chlorogenic acid‑related components of *Lonicera macranthoides* under different fertilization treatments. Environmental variables (red arrows): AP (available phosphorus), pH, TP (total phosphorus), and TK (total potassium). Response variables (blue arrows): CGA (chlorogenic acid), NCGA (neochlorogenic acid), CCGA (cryptochlorogenic acid), ICGA‑A (isochlorogenic acid A), ICGA‑B (isochlorogenic acid B), ICGA‑C (isochlorogenic acid C), FW (fresh weight), and DW (dry weight). Treatments are distinguished by distinct symbols/colors as indicated in the legend (● CK, ▲ T1, ■ T2, ◆ T3). Arrow length indicates the contribution of each variable to the ordination; arrows pointing in similar directions denote positive correlations. The first two constrained axes (RDA1 and RDA2) explained 57.9% and 37.8% of the total constrained variance, respectively (both axes were significant by permutation test, 999 permutations, *p* < 0.05). Abbreviations: CK = organic fertilizer + compound fertilizer; T1 = organic fertilizer + compound fertilizer + bacterial consortium (*Paenibacillus polymyxa*, *Bacillus amyloliquefaciens*, *Bacillus subtilis*, *Bacillus velezensis*, and *Bacillus nematocida*); T2 = organic fertilizer + compound fertilizer + simplified bacterial combination (*Paenibacillus polymyxa*, * Bacillus amyloliquefaciens*, and *Bacillus subtilis*); T3 = organic fertilizer + compound fertilizer + fungal agent (*Arbuscular mycorrhiza*, *Trichoderma harzianum*, and *Trichoderma longibrachiatum*).

As shown in Figure 3, redundancy analysis (RDA) was performed to further evaluate the joint relationships between soil factors, biomass production, and chlorogenic acid-related compounds in *Lonicera macranthoides*. The first two constrained axes explained 57.9% and 37.8% of the total constrained variance, respectively, indicating that the selected soil variables captured most of the variation in biomass and metabolite profiles among treatments. The ordination pattern showed a clear separation among fertilization treatments, suggesting that different microbial fertilizer regimes markedly altered the coupling relationships between soil conditions and plant responses. In the ordination space, AP and pH were oriented in a similar direction to FW, DW, and CGA, indicating that increased phosphorus availability and alleviation of soil acidity were closely associated with biomass accumulation and chlorogenic acid enrichment. By contrast, TP and TK were more closely aligned with ICGA-A, ICGA-B, and ICGA-C, suggesting that phosphorus and potassium pools contributed more strongly to the accumulation pattern of isochlorogenic acid components. NCGA and CCGA were positioned in a direction opposite to pH, implying that these compounds responded differently to soil chemical improvement compared with CGA.

## 4. Discussion

In this one-season field study, microbial fertilizer treatments altered the chemical properties of acidic soil, with the strongest responses observed at the pre-flowering stage. Compared with CK, T1 and T3 increased soil organic carbon, total phosphorus, available phosphorus, total nitrogen, hydrolyzable nitrogen, and pH. These observations are consistent with reports that functional microorganisms can increase labile phosphorus pools through organic acid production, phosphatase activity, and changes in the rhizosphere interface [22]. However, the present study did not directly measure microbial colonisation, microbial activity, phosphatase activity, organic acid secretion, or rhizosphere microbial community shifts. Therefore, the results should be interpreted as preliminary field observations that microbial fertiliser application was associated with higher phosphorus availability and reduced soil acidity, rather than as direct proof of a specific phosphorus-solubilising mechanism.

Specifically, *Bacillus* and *Paenibacillus* species are well-documented phosphate solubilizers that secrete organic acids (such as gluconic acid and citric acid) and extracellular phosphatases, which can convert insoluble phosphorus into plant-available orthophosphate [23,24]. *Trichoderma* spp. can acidify the rhizosphere through organic acid production and may enhance root proliferation via mycoparasitic or plant-growth-promoting mechanisms [25]. Arbuscular mycorrhizal fungi are known to extend the phosphorus uptake interface beyond the root depletion zone by means of extraradical hyphae. The stronger responses in T1 and T3 than in T2 may reflect functional complementarity among the inoculated microorganisms. But this interpretation remains a hypothesis requiring microbial and biochemical verification. Correlation analysis showed that available phosphorus was strongly positively correlated with fresh weight and dry weight, and that pH was positively correlated with chlorogenic acid, consistent with the view that phosphorus availability and acidity amelioration are central links connecting soil processes and plant metabolism [26]. Regression analysis indicated that available phosphorus explained a large proportion of the variation in total chlorogenic acid content (*R*^2^ = 0.727), further supporting the important role of phosphorus biotransformation in secondary metabolite accumulation [27]. In addition, T3 maintained the highest levels of total nitrogen and total potassium in July, which may be related to the characteristics of the fungal agent in long-term nutrient supply [28].

The observation that T1 outperformed T2 suggests that the two additional strains (*B. velezensis* ACCC 11067 and *B. nematocida* ACCC 11078) contributed functional traits not fully compensated by the three strains in T2. However, this interpretation remains speculative without dedicated experiments to isolate individual and combinatorial contributions of each strain.

Soil aggregate composition is an important indicator for evaluating soil structural stability [29]. In this study, microbial fertilizer treatments increased the proportion of >5 mm water-stable macroaggregates and decreased the proportions of some smaller fractions, indicating a shift towards larger water-stable aggregate classes. This pattern is consistent with the reported roles of microbial extracellular polysaccharides, organic binding agents, and fungal hyphae in aggregate formation [30]. The T3 treatment showed a relatively strong response in the >5 mm and 2–5 mm fractions, which may be related to fungal hyphal networks. Because extracellular polymeric substances and hyphal abundance were not quantified in this experiment, this explanation should be treated as a plausible mechanism rather than a demonstrated process [31,32]. The observed improvement in water-stable aggregates may have provided a more *favourable* physical environment for water retention and nutrient supply, which could contribute indirectly to biomass accumulation and metabolite formation in *L. macranthoides* [33].

Compared with water-stable aggregates, the effects of microbial fertilizer treatments on non-water-stable aggregates were more limited. Only in the >5 mm fraction did T3 show significantly higher values than CK in May; no significant differences were observed for most other fractions or treatments. This contrast suggests that the structural response to microbial fertilizer was more apparent in water-stable aggregates than in dry-sieved aggregate fractions [30,34]. For the 0.25–0.5 mm fraction, CK was significantly higher than T3 in both May and July, which may reflect a shift from smaller fractions to larger water-stable aggregates under microbial fertilizer treatment [35,36]. Overall, the influence of microbial fertilizers on non-water-stable aggregates is limited, and their ecological effects are mainly reflected in the formation of water-stable aggregates and the enhancement of soil erosion resistance.

The biomass and metabolite results indicate that microbial fertilizer treatments, particularly T1 and T3, were associated with improved production performance and changes in selected chlorogenic-acid-related compounds in *L. macranthoides*. T1 produced the highest fresh weight and dry weight and the highest numerical chlorogenic acid content, whereas T3 was more favourable for some isochlorogenic acid components. This pattern was broadly consistent with the treatment effects on available phosphorus and pH, supporting the working hypothesis that phosphorus availability and acidity status are important soil correlates of biomass and metabolite accumulation [37,38].

Regarding the plant-level response, the observed increases in chlorogenic acid and related compounds may be explained by improved phosphorus supply (enhancing ATP for the phenylpropanoid pathway) and pH moderation (affecting the micronutrient cofactor bioavailability for PAL and other enzymes) [39]. However, this mechanistic interpretation remains inferential rather than demonstrated.

However, the correlation, regression, and RDA results are associative. They support a putative nutrient- and acidity-associated pathway, but they do not establish that microbial fertilizers directly regulated chlorogenic acid biosynthesis. Different chlorogenic acid isomers also responded differently to soil variables: isochlorogenic acids A and C were positively correlated with total phosphorus and total potassium, whereas neochlorogenic acid and cryptochlorogenic acid were negatively correlated with pH. These findings suggest that microbial fertilizer regimes may alter not only total phenolic acid accumulation but also the allocation pattern among chlorogenic acid derivatives [40]. Future studies combining metabolite profiling with root traits, enzyme activity, microbial community sequencing, and rhizosphere exudate analysis will be needed to test this mechanistic interpretation.

Several limitations should be considered when interpreting these results. First, the experiment was conducted in a single year at one field site, so the generalisability of the treatment effects to other conditions remains uncertain. Multi-year and multi-site trials are required. Second, the study did not directly quantify microbial survival, colonization, enzyme activity, organic acid secretion, or rhizosphere community succession. Third, the statistical analysis involved multiple response variables with three replicate plots per treatment; therefore, individual significant associations should be interpreted cautiously and viewed as hypotheses for targeted validation. Fourth, whether the observed treatment effects were directly attributable to the introduced microorganisms, to shifts in the native microbiome, or to interactions between them remains unresolved. Future studies should incorporate cultivation-dependent and cultivation-independent approaches (e.g., plate counting, qPCR, and amplicon sequencing) to track inoculant persistence and community-level responses. These limitations do not negate the observed treatment responses, but they define the boundary between directly supported field findings and proposed mechanistic explanations.

## 5. Conclusions

This study provides preliminary indications that microbial fertilizer treatments differed in their associations with phosphorus availability, soil acidity, aggregate structure, biomass production, and chlorogenic-acid-related compounds in field-grown *L. macranthoides*. The bacterial consortium treatment (containing five strains: *Paenibacillus polymyxa*, *Bacillus amyloliquefaciens*, *Bacillus subtilis*, *Bacillus velezensis*, and *Bacillus nematocida*) and the fungal agent treatment (containing *Arbuscular mycorrhiza*, *Trichoderma harzianum*, and *Trichoderma longibrachiatum*) increased soil available phosphorus and pH and increased the proportion of water-stable macroaggregates, whereas the simplified bacterial combination (containing three strains: *Paenibacillus polymyxa*, *Bacillus amyloliquefaciens*, and *Bacillus subtilis*) had weaker effects. The bacterial consortium and the fungal agent also increased fresh and dry biomass; the bacterial consortium showed the highest numerical chlorogenic acid content, whereas the fungal agent was more favourable for isochlorogenic acids A and C. Available phosphorus and pH were the soil factors most closely associated with biomass and chlorogenic acid responses. These findings support the agronomic potential of microbial fertilizers in acidic *L. macranthoides* production, but the proposed nutrient- and acidity-associated pathways remain inferential. Multi-site and multi-year experiments that include microbial colonization, phosphatase activity, organic acid secretion, root traits, and microbial community dynamics are required to validate the mechanisms and broaden the applicability of the conclusions.

## Figures and Tables

**Table 1:** Temporal dynamics of soil chemical properties under different microbial fertilizer treatments.

Items	Month	Treatment				SEM	*p*-Value			
		CK	T1	T2	T3		*p* ANOVA	*p* (Time)	*p* (Treatment)	*p* (Time × Treatment)
Organic Carbon (g/kg)	Mar	16.92 b	18.30 ab	19.20 a	18.68 ab	0.618	0.039	<0.001	<0.001	<0.001
	May	19.69 c	69.63 a	49.65 b	69.16 a	2.861	<0.001			
	Jul	18.28 c	31.29 b	34.99 a	37.36 a	1.69	<0.001			
Total Potassium (g/kg)	Mar	16.22	15.70	17.03	18.97	0.035	0.893	0.087	0.002	0.156
	May	19.78 a	11.38 c	16.83 b	16.86 b	0.231	<0.001			
	Jul	15.76 b	16.24 ab	16.62 a	18.51 a	0.276	<0.001			
Total Phosphorus (g/kg)	Mar	0.53 a	0.30 b	0.45 ab	0.45 ab	0.77	0.089	<0.001	<0.001	<0.001
	May	1.45 c	8.10 a	2.70 b	7.67 a	0.752	<0.001			
	Jul	1.56 c	2.10 c	2.31 b	4.03 a	0.58	0.017			
Hydrolyzable Nitrogen (mg/kg)	Mar	164.90	160.78	262.37	160.00	0.051	0.02	0.052	<0.001	0.063
	May	188.53 c	380.60 a	356.75 b	378.18 a	0.834	<0.001			
	Jul	143.87 c	262.32 b	317.44 a	330.63 a	0.432	<0.001			
Available Phosphorus (mg/kg)	Mar	65.88 a	63.06 a	28.53 b	65.34 a	17.202	0.059	<0.001	<0.001	<0.001
	May	94.03 c	350.40 a	302.84 b	407.29 a	24.18	<0.001			
	Jul	60.68 c	174.68 a	116.78 b	195.27 a	31.072	<0.001			
Available Potassium (mg/kg)	Mar	191.97	196.83	123.80	198.10	6.581	<0.001	0.068	<0.001	0.011
	May	197.38 c	763.62 a	640.51 b	620.40 b	48.921	<0.001			
	Jul	175.30 c	332.34 b	325.72 b	425.84 a	24.384	<0.001			
Total Nitrogen (mg/kg)	Mar	1519.59	1483.22	1497.23	1470.49	12.438	0.068	<0.001	<0.001	<0.001
	May	1705.44 c	6371.75 a	4792.52 b	6447.14 a	73.642	<0.001			
	Jul	1411.29 c	2702.89 b	2988.94 b	3211.23 a	33.057	<0.001			
pH	Mar	4.67	4.92	4.70	4.54	0.073	0.138	<0.001	<0.001	<0.001
	May	4.35 c	6.48 a	6.16 b	6.40 a	0.212	<0.001			
	Jul	4.52 c	6.06 a	5.94 b	6.00 ab	0.212	<0.001			

Different letters within the same row indicate significant differences (*p* < 0.05). CK: organic fertilizer + compound fertilizer. T1: organic fertilizer + compound fertilizer + biological bacterial fertilizer. T2: organic fertilizer + compound fertilizer + *Bacillus polymyxa* + *Bacillus amyloliquefaciens* + *Bacillus subtilis*. T3: organic fertilizer + compound fertilizer + fungi (*Arbuscular mycorrhiza*, *Trichoderma harzianum*, *Trichoderma longibrachiatum*).

**Table 2:** Effects of different microbial fertilizer treatments on soil water-stable aggregates.

Size Fraction	Month	Treatment				SEM	*p*-Value			
		CK	T1	T2	T3		*p* ANOVA	*p* (Time)	*p* (Treatment)	*p* (Time × Treatment)
>5 mm	Mar	17.74	16.12	19.75	21.24	1.157	0.089	<0.001	<0.001	<0.001
	May	12.98 c	17.45 ab	16.15 bc	18.63 a	0.858	<0.001			
	Jul	10.93 b	14.54 a	13.22 ab	16.78 a	0.896	0.003			
2–5 mm	Mar	6.74 b	8.12 a	7.45 ab	7.63 ab	0.437	0.027	<0.001	<0.001	0.004
	May	7.97 b	9.70 a	9.10 ab	7.00 b	0.607	0.001			
	Jul	6.01 b	7.94 a	7.88 a	8.94 a	0.598	0.001			
1–2 mm	Mar	2.67	3.39	2.78	2.86	0.221	0.246	<0.001	<0.001	0.046
	May	5.76 a	3.82 b	4.40 b	2.82 c	0.358	<0.001			
	Jul	2.97 b	4.07 a	4.02 a	3.24 b	0.216	<0.001			
0.5–1 mm	Mar	7.03	7.29	6.29	6.60	0.329	0.25	<0.001	0.007	0.122
	May	8.15	7.00	7.17	7.71	0.379	0.214			
	Jul	7.73	8.23	8.53	7.34	0.303	0.076			
0.25–0.5 mm	Mar	4.89	3.99	3.79	2.98	0.288	0.054	<0.001	<0.001	<0.001
	May	5.28 a	3.01 b	3.55 b	3.66 b	0.392	0.002			
	Jul	6.97 a	4.67 b	4.90 b	3.18 c	0.443	<0.001			
0.15–0.25 mm	Mar	2.92	2.53	2.82	2.55	0.109	0.088	<0.001	<0.001	<0.001
	May	3.13 a	1.96 b	2.25 b	2.09 b	0.143	<0.001			
	Jul	4.00 a	2.74 b	3.00 b	2.42 b	0.199	<0.001			
<0.15 mm	Mar	8.01	8.56	7.11	6.14	0.578	0.086	<0.001	0.008	0.001
	May	6.49 b	7.06 ab	7.38 a	8.09 a	0.386	0.025			
	Jul	11.38 a	7.82 b	8.45 b	8.10 b	0.722	0.001			

Different letters within the same row indicate significant differences (*p* < 0.05). CK: organic fertilizer + compound fertilizer. T1: organic fertilizer + compound fertilizer + biological bacterial fertilizer. T2: organic fertilizer + compound fertilizer + *Bacillus polymyxa* + *Bacillus amyloliquefaciens* + *Bacillus subtilis*. T3: organic fertilizer + compound fertilizer + fungi (*Arbuscular mycorrhiza*, *Trichoderma harzianum*, *Trichoderma longibrachiatum*).

**Table 3:** Effects of different microbial fertilizer treatments on soil non-water-stable aggregates.

Items	Month	Treatment				SEM	*p*-Value			
		CK	T1	T2	T3		*p* ANOVA	*p* (Time)	*p* (Treatment)	*p* (Time × Treatment)
>5 mm	Mar	23.07	22.21	24.08	23.47	0.681	0.401	<0.001	0.047	0.011
	May	15.59 b	17.17 ab	16.44 ab	17.27 a	0.565	0.049			
	Jul	16.66	15.70	17.65	18.12	0.782	0.326			
2–5 mm	Mar	11.58	12.38	11.36	11.73	0.335	0.306	<0.001	0.176	0.102
	May	12.94	12.85	12.79	13.04	0.186	0.883			
	Jul	12.93	13.31	12.75	13.09	0.229	0.532			
1–2 mm	Mar	5.60	5.87	5.68	6.03	0.195	0.689	<0.001	0.537	0.007
	May	8.63 a	7.32 b	8.60 a	7.87 ab	0.254	0.002			
	Jul	7.95	8.44	7.79	7.52	0.24	0.174			
0.5–1 mm	Mar	4.31	4.52	4.12	4.08	0.124	0.17	<0.001	0.854	0.027
	May	6.54	5.82	6.59	6.05	0.18	0.066			
	Jul	6.30	6.21	5.82	5.66	0.213	0.267			
0.25–0.5 mm	Mar	2.58	2.56	2.32	2.25	0.078	0.064	<0.001	0.263	<0.001
	May	3.61 a	3.29 ab	3.15 b	3.00 b	0.106	0.007			
	Jul	3.39 a	3.31 a	3.10 ab	2.91 b	0.132	0.04			
0.15–0.25 mm	Mar	0.92	0.87	0.81	0.79	0.026	0.064	<0.001	0.131	0.003
	May	1.10	1.65	0.98	1.00	0.143	0.058			
	Jul	1.07	1.08	1.00	1.00	0.044	0.613			
<0.15 mm	Mar	1.93	1.59	1.64	1.65	0.063	0.054	<0.001	0.318	0.003
	May	1.58 b	1.91 ab	1.46 b	1.77 ab	0.101	0.049			
	Mar	1.70	1.950	1.90	1.70	0.086	0.183			

Different letters within the same row indicate significant differences (*p* < 0.05). CK: organic fertilizer + compound fertilizer. T1: organic fertilizer + compound fertilizer + biological bacterial fertilizer. T2: organic fertilizer + compound fertilizer + *Bacillus polymyxa* + *Bacillus amyloliquefaciens* + *Bacillus subtilis*. T3: organic fertilizer + compound fertilizer + fungi (*Arbuscular mycorrhiza*, *Trichoderma harzianum*, *Trichoderma longibrachiatum*).

**Table 4:** Responses of *Lonicera macranthoides* biomass and chlorogenic acid components to different microbial fertilizer treatments.

Items	Treatment				SEM	*p*-Value
	CK	T1	T2	T3		
Chlorogenic Acid	54.41	65.66	54.70	56.01	2.156	0.085
Neochlorogenic Acid	5.42 b	6.19 ab	6.60 a	6.56 a	0.242	0.041
Cryptochlorogenic Acid	4.05	3.88	4.23	4.07	0.412	0.711
Isochlorogenic Acid A	18.57 b	26.76 a	20.06 b	23.45 a	1.186	0.001
Isochlorogenic Acid B	2.22	2.29	2.21	2.35	0.085	0.859
Isochlorogenic Acid C	12.99 b	17.32 a	14.55 ab	15.44 ab	0.921	0.031
Isoquercitrin	0.61	0.54	0.80	0.74	0.055	0.380
Astragalin	0.46	0.47	0.52	0.49	0.023	0.488
Fresh Weight	431.23 b	888.88 a	393.41 b	701.98 a	67.890	0.034
Dry Weight	92.37 b	245.32 a	103.34 b	161.93 a	18.880	0.019

Different letters within the same row indicate significant differences (*p* < 0.05). CK: organic fertilizer + compound fertilizer. T1: organic fertilizer + compound fertilizer + bacterial consortium (*Paenibacillus polymyxa*, *Bacillus amyloliquefaciens*, *Bacillus subtilis*, *Bacillus velezensis*, and *Bacillus nematocida*). T2: organic fertilizer + compound fertilizer + simplified bacterial combination (*Paenibacillus polymyxa*, *Bacillus amyloliquefaciens*, and *Bacillus subtilis*). T3: organic fertilizer + compound fertilizer + fungal agent (Arbuscular mycorrhiza, *Trichoderma harzianum*, and *Trichoderma longibrachiatum*).

**Table 5:** Correlation analysis between soil nutrients and quality and yield of *Lonicera macranthoides.*

Items	Chlorogenic Acid	Neochlorogenic Acid	Cryptochlorogenic Acid	Isochlorogenic Acid A	Isochlorogenic Acid B	Isochlorogenic Acid C	Isoquercitrin	Astragalin	Fresh Weight	Dry Weight
Different fertilization treatments	0.707 *	−0.593 *	−0.698 *	0.395	−0.121	−0.019	0.11	0.13	0.608 *	0.607 *
Organic carbon	0.423	−0.294	−0.475	0.533	0.028	0.166	0.252	0.175	0.603 *	0.604 *
Total nitrogen	0.418	−0.243	−0.463	0.504	0.041	0.134	0.359	0.188	0.612 *	0.619 *
Total potassium	−0.047	0.278	0.051	0.836	0.535	0.600 *	0.315	−0.205	0.55	0.556
Total phosphorus	−0.005	0.261	0.138	0.865	0.572	0.624 *	0.281	−0.007	0.516	0.494
Hydrolyzable nitrogen	0.299	−0.342	−0.553	0.449	−0.04	−0.074	0.227	−0.193	0.408	0.434
Available phosphorus	0.538	−0.2	−0.332	0.528	0.371	0.47	−0.189	0.146	0.804	0.781
Available potassium	0.372	−0.143	−0.145	0.601 *	0.246	0.377	0.138	0.203	0.496	0.469
pH	0.687 *	−0.607 *	−0.681 *	0.338	−0.109	−0.062	−0.013	0.177	0.559	0.549

Note: * p < 0.05. Values without asterisks are not statistically significant.

**Table 6:** Regression analysis of soil nutrients on yield and quality of *Lonicera macranthoides*.

Dependent Variable	Predictor Variable	Regression Equation	*R* ^2^	Adjusted *R*^2^	F-Value	Coefficient (B)	*p*-Value
Fresh weight	SOC	y = 27.51 · SOC	0.364	0.300	5.722	27.512	0.038
	TN	y = 249.50 · TN	0.374	0.311	5.975	249.503	0.035
	AP	y = 5.03 · AP	0.646	0.611	18.260	5.030	0.002
Dry weight	SOC	y = 6.74 · SOC	0.365	0.301	5.740	6.740	0.038
	TN	y = 61.84 · TN	0.384	0.322	6.223	61.839	0.032
	AP	y = 1.20 · AP	0.610	0.571	15.626	1.196	0.003
Total chlorogenic acid	SOC	y = 0.36 · SOC	0.392	0.331	6.443	0.356	0.029
	TN	y = 3.09 · TN	0.370	0.307	5.877	3.093	0.036
	TP	y = 2.69 · TP	0.346	0.281	5.299	2.689	0.044
	AP	y = 0.07 · AP	0.727	0.700	26.686	0.067	0.000
	AK	y = 0.03 · AK	0.501	0.451	10.051	0.031	0.010
	pH	y = 4.99 · pH	0.512	0.451	8.383	4.989	0.016

Note: Abbreviations: SOC: Soil organic carbon; TN: Total nitrogen; TP: Total phosphorus; AP: Available phosphorus; AK: Available kalium/potassium.

## Data Availability

The data that support the findings of this study are available from the corresponding author upon reasonable request.
